# Bromide of Ethyl

**Published:** 1880-07

**Authors:** 


### ARTICLE IV.

### *Bromide of Ethyl.*

The properties and value of this new anæsthetic were quite extendedly discussed at a recent meeting of the Paris Surgical Society. Half a dozen gentlemen related their experience, which, on the whole, gave a qualified endorsement of the drug. The same characteristics, good and bad, which have already been noticed by American surgeons, were observed by the French. No fatal cases were reported however.

Mr. Verneuil was struck with the rapid and powerful action of the anæsthetic. One of his patients, a woman, to whom he gave it, was put to sleep almost in an instant. M. Berger was also impressed with its power; a rabbit put under a bell-glass, in which were placed ten grammes (ʒ iiss.) of bromide of ethyl, expired at once. He had never seen so rapid an action in the case of chloroform or ether. These illustrations may be remembered in connection with the general fact, asserted by Dr. Turnbull, that the danger of an anæsthetic is in proportion to its strength of action. We may state here also the fact that a death is reported to have recently occurred in the practice of Dr. Levis while using the bromide. We are not informed, however, that the anæsthetic was the direct or sole cause of the death.

The opinion was expressed, during the discussion referred to, that the bromide of ethyl did not easily produce complete muscular relaxation, and could not be used in operations where such a condition is essential. The opinions, as we have said, while not against its use, were by no means strongly in its favor. This refers to the ether when inhaled. As a local anæsthetic it was spoken of highly. It is the only anæsthetic of this kind which, owing to its non-inflammability, can be safely used in connection with the actual cautery; such was the opinion of M. Micaise. Evidence of its value in this direction has been given by American surgeons, and the point was referred to by Dr. Turnbull, at the recent meeting of the American Medical Association.

It seems that bromide of ethyl is rapidly finding its true place as an anæsthetic, and that place is not a very high one.—*Medical Record.*

